# Optimization of Spectrum Utilization in Cooperative Spectrum Sensing

**DOI:** 10.3390/s19081922

**Published:** 2019-04-23

**Authors:** Mohsin Ali, Haewoon Nam

**Affiliations:** 1 Department of Computer Engineering, Khwaja Fareed University of Engineering and Information Technology (KFUEIT), Rahim Yar Khan 64200, Pakistan; mohsin.ali@kfueit.edu.pk; 2 Division of Electrical Engineering, Hanyang University, Ansan 15588, Korea

**Keywords:** cognitive radio, spectrum hole utilization, spectrum sensing, soft combination

## Abstract

This paper presents an analytical framework for the probability of spectrum hole utilization (PSHU) of a cognitive radio system with soft cooperative spectrum sensing (CSS) under a practical consideration of fixed frame structure. In practical systems, the length of a time-frame is generally fixed, where the time-frame consists of sensing, reporting, and transmission durations. Thus, increasing sensing and reporting time duration in cooperative spectrum sensing improves the probability of successful detection of the primary user’s (PU) presence or the absence but reduces transmission time duration, which results in a lower PSHU. A large reporting duration is required when more secondary users (SUs) report their sensed information to the fusion center (FC) and/or multiple bits are used by each SU in soft cooperative spectrum sensing. Thus, reporting time in terms of the number of SUs and reporting bits also have a similar effect on PSHU. Based on this interesting trade-off between PSHU and the sensing and reporting time duration, this paper analyzes the impact of an increasing number of SUs and reporting bits on PSHU.

## 1. Introduction

Cognitive radio (CR) is an emerging technology in wireless communication systems that has recently taken immense attention by the researchers [1]. The primary goal of CR is to allow secondary users (SUs) to have dynamic access to the radio frequency (RF) spectrum to increase spectrum utilization and resolve the problem of underutilized licensed frequencies [2]. Specifically, SUs need to detect the spectrum opportunities or spectrum holes that are not actively used by primary users (PUs) and then take advantage of them for communications with other SUs until releasing them to PUs upon their reappearance in the frequency band [3,4]. Spectrum hole utilization is considered advantageous only under the condition that SUs communicate with each other without interfering with the PUs’ transmission. A single SU may not be able to detect the spectrum hole reliably and correctly due to transmission impairments like shadowing and multipath fading [5]. Missed detection can degrade CR systems’ performance, specifically regarding the probability of spectrum hole utilization (PSHU). Thus, cooperative spectrum sensing (CSS) has attracted growing interest from researchers due to the fact that a higher detection probability of spectrum holes can be achieved by the cooperation of multiple SUs [6,7,8].

In cooperative spectrum sensing, the operating procedure consists of the following four steps: (1) local sensing, (2) reporting, (3) decision and (4) transmission [9,10]. In the local sensing step, each SU senses the given spectrum and collects observations that could potentially capture the primary users’ signals. In the reporting step, SUs forward their observations or local decisions to the fusion center (FC). In the decision phase, FC makes a global decision about the presence or the absence of primary users based on the collected local observations by using a hard or a soft combining scheme [11,12,13,14]. The authors in [15] have shown the criteria for an effective decision approach selection analytically with high detection accuracy as an objective in the presence of realistic channel propagation effects for two different decision approaches as combining decision (CD) and sensing and combining decision (SCD). Different cooperative sensing techniques are implemented according to both decision approaches and, on the basis of detection accuracy of these techniques, authors have shown the selection threshold between the two approaches. In our proposed work, a centralized cognitive radio with multiple SUs and FC is considered, which is the same as the CD decision approach; a soft combination scheme is also considered. Thus, all SUs forward their sensing results to FC in multiple bits form and eventually FC makes the final decision about PU presence and absence. In our manuscript, we assumed the concept of equal gain combining (EGC) at FC in which all the received signal from SUs are weighted with the same factor, so FC will just combine the energy of all of the received signals and compare that with predefined detection threshold to decide the PU’s presence and absence. Authors in [16] have shown the effects of temporal dispersive reporting channel on cooperative spectrum sensing and two proposed fusion schemes, widely linear (WL) and linear (L) are compared with each other in the form of probabilities of detection and false alarm. In our proposed work, we assumed an ideal AWGN reporting channel and the effects of temporal dispersion on the reporting channel are also assumed to be neglected, which can be due to several factors, such as multiple SUs, multi-path propagation, etc. We assumed that all SUs are located around the FC in such a way that each SU has less movement and small distance with FC so that the temporal dispersive reporting channel can be avoided due to multi-path prorogation delay. For multiple SUs, we assumed that all SUs report their data to FC in a time division multiple access (TDMA) way.

If an active primary user is not observed, namely a spectrum hole is detected, SUs can communicate with each other in the last transmission phase. Spectrum utilization in CR systems are often characterized by the probability of spectrum hole utilization (PSHU), which is defined as the successful data transmission by the secondary users (SUs) in the given transmission time provided that spectrum hole (primary user’s absence in the spectrum) is correctly declared and there is no interference to primary users (PUs) during the transmission [17,18,19,20].

There is an intriguing trade-off between the sensing and reporting durations and spectrum utilization. Increasing sensing time duration improves the probability of successful detection of the presence or the absence of PUs but reduces transmission time duration, resulting in a lower PSHU. On the contrary, reducing sensing time duration allows a large transmission time duration but SUs’ transmission may cause interference to PUs due to a lower probability of successful detection of PUs’ presence or the absence. Similarly, increasing the number of SUs in CSS and/or using a large number of bits for reporting enhances detection probability of PUs at the FC but requires longer reporting time, which reduces transmission time and further spectrum utilization. However, decreasing the number of SUs may lead to a higher miss-detection of PUs.

Most of the existing papers discuss the effect of varying numbers of secondary users and the fixed time frame structure on cognitive radio networks’ throughput, but, in our paper, the main point of discussion is the probability of spectrum hole utilization (PSHU). Existing papers consider a hard fusion scheme for cooperative spectrum sensing to calculate the probability of spectrum hole utilization and they did not consider the effect of the number of reporting bits on PSHU, as in a hard fusion scheme each SU reports its data to fusion center about primary users’ presence or absence in 1-bit form. In our paper, as we consider the soft fusion scheme for cooperative spectrum sensing to calculate the PSHU, so the effect of both the number of SUs and reporting bits is shown on PSHU. Thus, to the best of our knowledge, the joint optimization of the number of SUs and reporting bits has not been discussed to maximize the probability of spectrum hole utilization for cooperative spectrum sensing under a soft fusion scheme.

To the best of our knowledge, no work has been reported to properly study the impact of the number of SUs and the number of bits in reporting on the probability of spectrum hole utilization under the soft data fusion scheme. Thus, it is meaningful to investigate the effect of increasing sensing time to achieve low false alarm probability, denoted by $P_{f}$ and increasing reporting time due to the number of SUs and the reporting bits on the spectrum utilization under a practical consideration of a fixed frame structure.

The main contributions of this paper are summarized as follow: First, a CR system with a fixed frame structure is proposed and the impacts of the number of SUs and the number of bits for reporting on PSHU in the system are analysed. Simulation results support the derived analytical expression for PSHU. Second, it is shown by simulations that the optimal number of SUs can be achieved for the maximum PSHU given that the number of bits for reporting is fixed. Similarly, the optimal number of bits for reporting can also be obtained while the optimal number of SUs is fixed. Furthermore, we jointly optimize the number of secondary users (SUs) and reporting bits to maximize the probability of spectrum hole utilization (PSHU) of our proposed system. The effect of the number of SUs and reporting bits on reporting and transmission time in a fixed time frame of SUs is analysed to maximize the PSHU. Thus, an analytical analysis of the optimal number of SUs and reporting bits to maximize the PSHU for cooperative spectrum sensing under soft fusion scheme is provided. We also provide the simulation results in support of our analytical analysis.

## 2. Related Works

In this section, some of the related works in the literature are discussed. The probability of successful joint idle channel detection and packet transmission, which is defined as the utilization of SOP (USOP), is discussed in [19] and determined the probability of USOP in a dynamic radio environment under imperfections in the channel sensing for different network topologies. Authors considered the fixed transmission time for secondary users (SU), but they did not consider the effect of sensing and reporting time duration on probability of USOP. In [20], a trade-off between spectrum sensing and the probability of spectrum opportunity utilization is shown. Authors investigated the optimal sensing time considering both spectrum sensing performance and the utilization of spectrum opportunity. Authors have considered that, when sensing time increases transmission time also increases, a variable time frame is considered, which is not a practical scenario.

In [21], a new method is proposed for adapting the size of the spectrum sensing window that improves the detection of the spectrum holes in dynamic scenarios along with their actual utilization. Authors have investigated the utilization of spectrum holes in cognitive radio (CR) systems with energy-based spectrum sensing and defined a formal measure for spectrum hole utilization which is used to analyze how the size of the sensing window, signal-to-noise ratio (SNR) and PU activity affect the utilization of spectrum holes.

Authors in [22] formulated and analysed the sum utilization of spectrum with re-transmissions for the SU to ensure the reliable packet delivery under different PU and SU co-existing network topologies. SU’s packet transmission reliability is considered by allowing SU to re-transmit its packet during the idle state of PU in case of packet loss. The sum utilization of spectrum is analysed individually for different scenarios of transmissions, i.e., without re-transmission, and with the different number of re-transmissions, under both spatial and temporal variations for the PU activity. The spectrum utilization trade-off between different sensing and PUs’ mean idle durations, under certain conditions, has been investigated. Optimal sensing time has been calculated to maximize spectrum utilization and the performance of spectrum utilization of SU is also investigated for each re-transmission of the SU to ensure the reliable packet delivery for each network topology.

A spectrum allocation model that maximizes the spectrum utilization, based on the interference among primary and secondary users by providing the spectrum allocation solution for a cognitive radio network (CRN), is proposed in [23]. Authors have developed an enhanced artificial bee colony algorithm called the Modified Binary ABC (MBABC) algorithm to solve spectrum allocation problem. In the MBABC algorithm, each possible spectrum assignment solution is encoded as a bit string. A solution pool is selected according to different selection pressure schemes. New solutions are produced by applying mutation and crossover operations. An acceptable allocation solution is achieved by conducting a series of evolution cycles. The simulation results of MBABC are discussed and compared with the results of existing methods such as Binary ABC (BABC), Memetic ABC (MemABC) and a random method (RAND). Authors claim that MBABC method outperforms the other methods in terms of an efficient data transmission and QoS; it also provides the better allocation solution to maximize the spectrum utilization.

Authors in [24] investigated the cognitive transmissions with multiple relays by jointly considering the spectrum sensing and data transmissions phases over Rayleigh fading channels. Two transmission schemes for multiple-relay cognitive radio networks are explained. The first one is selective fusion spectrum sensing and best relay data transmission scheme (SFSS-BRDT) and the second one is fixed fusion spectrum sensing and best relay data transmission scheme (FFSS-BRDT). Closed-form expressions of the spectrum hole utilization efficiency for the two schemes have been derived under the Rayleigh fading channels. It is shown that SFSS-BRDT scheme outperforms the FFSS-BRDT scheme in terms of the spectrum hole utilization efficiency. The effect of increasing number of CRs and spectrum sensing overhead in both schemes to find out the spectrum hole utilization efficiency is also shown.

To reduce the overall sensing overhead, three spectrum sensing strategies have been developed in [25], which adapt the scheduling of sensing slot and sensing cycle to the dynamic network environments. The proposed interference avoidance (IA) strategy and interference control (IC) strategies can be either used individually or combined to meet the system requirements and the medium access control protocol for SUs. Sensing parameters are utilized and optimized to improve the overall spectrum utilization efficiency under interference restrictions.

In [26], a resource allocation scheme is proposed to maximize the long-term network-level throughput in energy-constrained cooperative cognitive radio networks (CCRNs) by considering the user diversity of SUs in channel condition, traffic load and energy amount. Spectrum utilization and network-level throughput for secondary networks are improved by allowing all SUs to optimally share the cooperation-generated period, and jointly formulate the relay selection, secondary transmission scheduling, and power allocation problems. A resource allocation problem is formulated under the energy limitations of SUs and an online spectrum utilization maximization (SUM) scheme is designed.

Authors in [27] proposed an energy-based interference signal detection framework for the Industrial, Scientific and Medical (ISM) radio band cognitive radio sensors networks (CRSNs) under multiple wireless technologies (e.g., WSNs, WiFi networks) to analyse the detection performance and spectrum utilization with respect to various parameters such as the sensing time, SNR, decision threshold, and the prior probability. An exact and approximate solution of the detection metrics is provided by using the hypothesis test and determined the spectrum utilization of the network.

Different methods are introduced in the above mentioned existing works to maximize the spectrum hole utilization by optimizing the different system parameters like, sensing time and transmission time, etc. and ensuring the minimum interference to PUs. However, most of the authors considered a dynamic time frame structure and fixed sensing and transmission time slots to calculate the probability of spectrum utilization, which is not a practical scenario; contrary to this, we have considered a practically used fixed time frame and dynamic sensing, reporting and transmission time slots in our work. Some authors also considered the cooperative spectrum sensing, but they did not show the effect of number of reporting bits on probability of spectrum hole utilization for soft cooperative spectrum sensing, but, in our work, we have considered the effect of both number of SUs and reporting bits on PSHU for soft cooperative spectrum sensing. We also showed the joint optimization of the number of SUs and number of reporting bits to maximize the probability of spectrum hole utilization, which lacks in the existing works.

## 3. System Model

**Figure 1:**
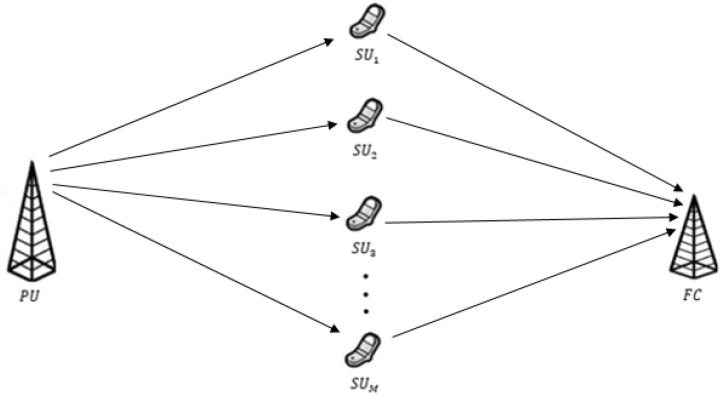
Illustration of a cognitive radio network.

A centralized CR network with *M* secondary users and an FC is considered as shown in Figure 1.

Assume that all SUs are independently and identically distributed (i.i.d.) such that they experience the same signal-to-noise ratio (SNR) of the sensing channel in the area of coverage of primary signal. For spectrum sensing, an energy detection (ED) technique is assumed due to its simple implementation and no need for prior knowledge of source signal and channel fading [28]. In the CR network, soft cooperative spectrum sensing is used, in which each SU collects a certain number of samples and then reports the average energy of the collected samples to FC in the form of multiple bits. The FC then combines the reported values from all SUs and makes the decision by comparing the combined value with a predefined detection threshold [29,30,31]. Another assumption is taken that all SUs report their information to FC through reporting channel in time division multiple access (TDMA) manner.

Received signal by SUs can be modelled as binary hypothesis and is expressed as

(1) $$y\left(s\right)=\left\{\begin{array}{cc} w(s), & H_{0},\\ x(s)+w(s), & H_{1}, \end{array}\right.$$

where s=1,2,…S is the sample index collected by SUs, *S* is the total number of collected samples for sensing, x(s) is primary user’s signal with variance $\sigma_{x}^{2}$ and w(s) is additive white Gaussian noise (AWGN) with zero mean and variance $\sigma_{w}^{2}$. $H_{0}$ and $H_{1}$ show the hypotheses for the absence and the presence of primary signal, respectively.

### 3.1. Frame Structure

**Figure 2:**
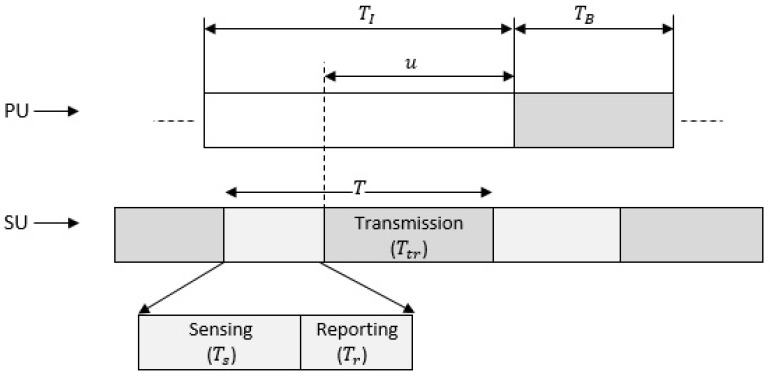
Primary user’s (PU) idle ($T_{I}$) and busy ($T_{B}$) time representation and Secondary users’ (SUs) time frame (*T*) structure.

Figure 2 illustrates the frame structure of the proposed CR network, where a time frame (*T*) for SUs is divided into three parts according to the sequence of operations done by SUs. The three parts of time frame are named as sensing time ($T_{s}$), reporting time ($T_{r}$) and transmission time ($T_{tr}$). The length of a time frame is expressed as $T=T_{s}+T_{r}+T_{tr}$. Reporting time ($T_{r}$) can vary depending on the number of SUs (*M*), the number of reporting bits (*N*) and the reporting channel sampling frequency ($f_{r}$). Thus, the reporting time can be expressed as $T_{r}=\frac{MN}{f_{r}}$ [29]. Thus, the time frame can now be expressed as

(2) $$T=T_{s}+\frac{MN}{f_{r}}+T_{tr}.$$

The idle and the busy time durations of PU are denoted by $T_{I}$ and $T_{B}$, respectively. The transition of PU from busy to the idle state, or vice versa, follows the Poisson process. As in the Poisson process, the occurrence of certain events happens at a certain rate but are completely random in nature. As we know that PU has two states, it will either be busy or idle in the spectrum, but the occurrence time of these states is completely random [32]. Thus, by looking at the behavior of PU’s state transition, it can be modeled as a Poisson process. Thus, both $T_{I}$ and $T_{B}$ can be modeled as exponential distributed with mean $\alpha_{I}$ and $\alpha_{B}$, respectively [33], and probability distribution functions (PDFs) as $f_{T_{I}}\left(t\right)=\frac{1}{\alpha_{I}}e^{-\frac{t}{\alpha_{I}}}$ and $f_{T_{B}}\left(t\right)=\frac{1}{\alpha_{B}}e^{-\frac{t}{\alpha_{B}}}$, respectively.

### 3.2. Practical Considerations

In most of the practical systems, the frame length is required to be fixed for various reasons such as time synchronization between a transmitter and a receiver. Thus, it is assumed throughout this paper that the length of a time frame *T* is fixed. However, within a given fixed time frame, the sensing duration or the reporting duration can be variable depending on the number of samples taken by an SU and the number of SUs in cooperative spectrum sensing. The larger number of samples collected for sensing, the longer sensing duration will be. Similarly, the greater the number of SUs that participate in the cooperative spectrum sensing and/or the larger number of bits that is used for reporting, the longer the reporting duration will be. Note that longer sensing and reporting durations result in less transmission time, which further leads towards a less utilization probability of spectrum. Thus, there is an interesting trade-off between the number of SUs and number of reporting bits versus spectrum utilization regarding PSHU. Note that PSHU is a probability of utilizing the idle spectrum band by SUs during the transmission slot of a given time frame before the reappearance of PU in that spectrum band. PSHU mainly depends upon correct detection of the absence of the primary signal and the length of transmission time for SUs. The higher correct detection probability and longer transmission time, the higher PSHU is generally expected.

## 4. Cooperative Spectrum Sensing with Soft Bits

In soft cooperative spectrum sensing, each SU collects (*S*) samples from the channel during the sensing process and reports the average energy of those samples to the FC using a quantized form of *N* bits. When the quantized average energy from all SUs are received, the FC combines those and compares it with a predetermined detection threshold (λ) in order to reach the final decision. Test statistics calculated at the FC are given as

(3) $$E=\frac{1}{MS}\sum_{m=1}^{M}\sum_{s=1}^{S}\left|y_{m}{\left(s\right)}^{2}\right|.$$

Assuming the number of samples and the number of users are large, applying a central limit theorem leads to the detection probability $\left(P_{d}\right)$ and the false alarm probability $\left(P_{f}\right)$ as [34]

(4) $$P_{f}=Pr\left(E>\lambda |H_{0}\right)=Q\left(\left(\frac{\lambda }{\sigma_{n}^{2}}-1\right),\sqrt{MS}\right)$$

(5) $$P_{d}=Pr\left(E>\lambda |H_{1}\right)=Q\left(\left(\frac{\lambda }{\sigma_{n}^{2}}-\gamma -1\right)\sqrt{\frac{MS}{2\gamma +1}}\right),$$

where γ is SNR and Q(·) is Q-function defined by

(6) $$Q\left(z\right)=\frac{1}{\sqrt{2\pi }}\int_{z}^{\infty }exp\left(-\frac{v^{2}}{2}\right)dv.$$

In practical systems, a certain level of detection probability often needs to be satisfied due to performance requirements. In case a target detection probability $\bar{P_{d}}$ is given, the detection threshold can be calculated from Equation (5) as

(7) $$\lambda =\sigma_{n}^{2}\left(\gamma +1+\sqrt{\frac{2\gamma +1}{MS}}Q^{-1}\left({\bar{P}}_{d}\right)\right).$$

Substituting Equation (7) into Equation (4), the probability of false alarm can be rewritten as a function of target detection probability as

(8) $$P_{f}=Q\left(\sqrt{2\gamma +1}Q^{-1}\left({\bar{P}}_{d}\right)+\gamma \sqrt{MS}\right).$$

## 5. Performance Analysis of PSHU under Soft Cooperative Spectrum Sensing

Based on the mean of idle and busy states of PU, the probabilities of being idle and busy can be calculated as [18]

(9) $$\theta_{I}=\frac{\alpha_{I}}{\alpha_{I}+\alpha_{B}},\;\theta_{B}=\frac{\alpha_{B}}{\alpha_{I}+\alpha_{B}}.$$

In cooperative spectrum sensing, SUs are allowed to utilize the spectrum band of interest, when FC correctly detects the idle state of PU with the probability $P_{CD}$, which can be expressed as

(10) $$P_{CD}=\left(1-P_{f}\right).$$

In soft cooperative spectrum sensing, $P_{f}$ calculated at FC decreases with increasing numbers of SUs. The number of bits (*N*) used in data reporting to FC also has an effect on $P_{f}$. When *N* increases, $P_{f}$ goes down because, by increasing the number of reporting bits, the number of quantization levels $\left(2^{N}\right)$ also increase, which helps to achieve low $P_{f}$. Thus, having a low $P_{f}$ helps in a higher probability of correct detection ($P_{CD}$) of primary users’ signal, which further helps SUs to utilize idle spectrum band more efficiently. Transmission of SUs is considered successful when it is done during the PU idle time, which requires PU idle duration to be greater than SUs’ transmission time—in other words, no reappearance of PU in the spectrum during the SUs’ transmission duration ($T_{tr}$). In this way, there is no interference by SUs into PU. This can be defined as the probability of no interference ($P_{NI}$), which is expressed as

(11) $$P_{NI}=Pr\left(u>T_{tr}\right),$$

where $T_{tr}$ is transmission time of SU as in Equation (2) and *u* is the duration of idle time of PU, which is assumed to start just after the SUs’ sensing time ($T_{s}$) and reporting time ($T_{r}$) until PU reappears in the spectrum. Since *u* can be modelled as exponentially distributed [33] with mean value $\alpha_{0}=\alpha_{I}-T_{s}-\frac{MN}{f_{r}}$ and its PDF of $f_{u}\left(t\right)=\frac{1}{\alpha_{0}}e^{-\frac{t}{\alpha_{0}}}$ as shown in the Figure 2, Equation (11) can be re-written as

(12) $$P_{NI}=\int_{T_{tr}}^{\infty }\frac{1}{\alpha_{0}}exp\left(-\frac{t}{\alpha_{0}}\right)dt=\exp\left(-\frac{T-T_{s}-\frac{MN}{f_{r}}}{\alpha_{0}}\right).$$

SUs’ transmission is only successful and the spectrum is fully utilized when all the following conditions are met:

1. PU is idle with the probability ($\theta_{I}$),
2. Correct detection of PU idle state or spectrum hole ($P_{CD}$),
3. PU does not reappear during the SUs’ transmission such that no interference occurs by SUs to PU ($P_{NI}$),

where $\theta_{I}$ is defined in Equation (9). Therefore, spectrum hole utilization takes place when SUs transmission is successful (all the above conditions are met) during the given transmission duration probability $\left(1-\frac{T_{s}}{T}-\frac{MN}{Tf_{r}}\right)$ of SUs. Calculation of PSHU in our paper differs from the utilization probability shown in other literature, in such a way that we consider the practical scenario of CRN in which fixed time frame of SUs is taken. Thus, in a fixed time frame, increasing the sensing and reporting time causes the decrease in transmission time. Sensing time increases achieving low $P_{f}$ and reporting time increases when the number of SUs involved in cooperative spectrum sensing increases and also due to increasing the number of reporting bits. Thus, PSHU can be defined by considering the above conditions and the given transmission duration and is expressed as

(13) $$\begin{array}{c} P_{SHU}=\left(1-\frac{T_{s}}{T}-\frac{MN}{Tf_{r}}\right)\theta_{I}P_{CD}P_{NI}\\ \;=\left(1-\frac{T_{s}}{T}-\frac{MN}{Tf_{r}}\right)\theta_{I}\left(1-P_{f}\right)\exp\left(-\frac{T-T_{s}-\frac{MN}{f_{r}}}{\alpha_{0}}\right). \end{array}$$

From Equation (13), it is clear that PSHU can be maximized by increasing transmission time in a fixed time frame and in a given PU’s idle time ($\alpha_{0}$). PSHU can also be increased by lowering down $P_{f}$. In soft cooperative spectrum sensing, low $P_{f}$ can be achieved by increasing the number cooperating users and reporting bits. Thus, by incorporating the effect of transmission time, the number of SUs and reporting bits, maximum PSHU can be achieved in a given scenario. Since the optimal number of SUs and the optimal number of bits for reporting that maximize PSHU in Equation (13) are difficult to compute with a simple closed form, numerous simulations are performed to show the optimal numbers versus PSHU. These results are shown in the following section.

To maximize the probability of spectrum hole utilization (PSHU), optimization problem can be written as

(14) $$\begin{array}{c} \max\;P_{SHU}\left(M,N\right),\\ \mathrm{subject}\;\mathrm{to}\;P_{d}\geq {\bar{P}}_{d},\\ 1<m\leq M,1<n\leq N. \end{array}$$

To find out the optimal value of *M* and *N*, a partial derivative of Equation (13) is taken w.r.to *M* and *N*, respectively. The first order partial derivative of Equation (13) w.r.t. *M* is expressed as

(15) $$\begin{array}{c} \frac{\partial P_{SHU}\left(M,N\right)}{\partial M}=\theta_{I}e^{\left(-\frac{T-T_{s}-\frac{MN}{f_{r}}}{\alpha_{0}}\right)}[\left(1-\frac{T_{s}}{T}-\frac{MN}{Tf_{r}}\right)\\ \;\times \left[\frac{N\left(\alpha_{I}-T\right)\left(1-P_{f}\right)}{f_{r}\alpha_{0}^{2}}-P_{f}^{\prime }\left(M\right)\right]-\frac{N}{Tf_{r}}\left(1-P_{f}\right)], \end{array}$$

where $P_{f}^{\prime }\left(M\right)=-\frac{\gamma \sqrt{S}}{2\sqrt{2\pi M}}\exp\left(-\frac{{\left(\sqrt{2\gamma +1}Q^{-1}\left({\bar{P}}_{d}\right)+\gamma \sqrt{MS}\right)}^{2}}{2}\right)$. From Equation (15), $\frac{\partial P_{SHU}}{\partial M}$ is a monotonically decreasing function of *M*, so $P_{SHU}\left(M,N\right)$ is a concave function and will have an optimal value of the number of SUs ($m^{*}$) over which maximum PSHU can be achieved.

Similarly, to find the optimal number of bits, the partial derivative of Equation (13) w.r.t. *N* is taken and is expressed as

(16) $$\begin{array}{cc} \frac{\partial P_{SHU}\left(M,N\right)}{\partial N} & =\theta_{I}\left(1-P_{f}\right)e^{\left(-\frac{T-T_{s}-\frac{MN}{f_{r}}}{\alpha_{0}}\right)}[\left(1-\frac{T_{s}}{T}-\frac{MN}{Tf_{r}}\right)\\ & \times \frac{M(\alpha_{I}-T)}{f_{r}\alpha_{0}^{2}}-\frac{M}{Tf_{r}}]. \end{array}$$

$\frac{\partial P_{SHU}}{\partial N}$ is also monotonically decreasing function of *N*, so $P_{SHU}\left(M,N\right)$ is concave function and will have an optimal number of reporting bits ($n^{*}$) to achieve the maximum PSHU. Thus, the optimization problem in Equation (14) can be rewritten as

(17) $$\begin{array}{c} \max\;P_{SHU}\left(M,N\right),\\ \mathrm{subject}\;\mathrm{to}\;1<m^{*}\leq M,\\ \;1<n^{*}\leq N. \end{array}$$

## 6. Numerical Results

The following values of system parameters are taken in simulations: T=20 ms, $f_{s}=1$ MHz, $\bar{P_{d}}=0.9$, $\alpha_{I}=650$ ms, $\theta_{I}=0.9$, γ=−10 dB, $f_{r}=10$ kHz. These parameters have influences on system performance and also have the ability to change the system and channel characteristic. Also according to the definition of PSHU, interference to PU is completely avoided by ensuring the correct detection of spectrum hole and by assuming that PU idle time is greater than SUs’ transmission duration. Ten thousand Monte Carlo simulations of $P_{f}$ are performed in Matlab 2018a (MathWorks, Inc., Natick, MA, USA) and these results are used to find out the numerical results of PSHU.

**Figure 3:**
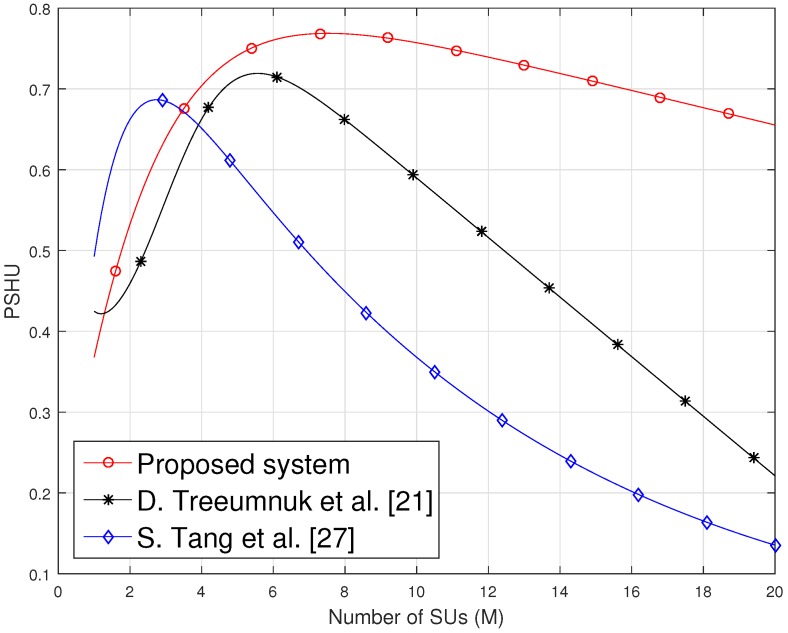
Probability of spectrum hole utilization (PSHU) vs. different number of SUs *(M)*.

Figure 3 shows PSHU of the proposed system as a function of the number of SUs (*M*) under soft cooperative spectrum sensing. Fixed number of reporting bits i.e., N=4 is considered for each SU and $P_{f}$ is calculated according to Equation (8) to analyze the effect of the number of SUs on PSHU. The result shows that increasing number of SUs is not always helpful to improve spectrum utilization. As the number of SUs increases, $P_{f}$ decreases, but reporting time of the system increases accordingly, which causes a reduction in the transmission time of SUs and further reduces the PSHU. Hence, the figure shows the optimal value of the number of SUs in cooperative spectrum sensing to achieve the maximum PSHU.

**Figure 4:**
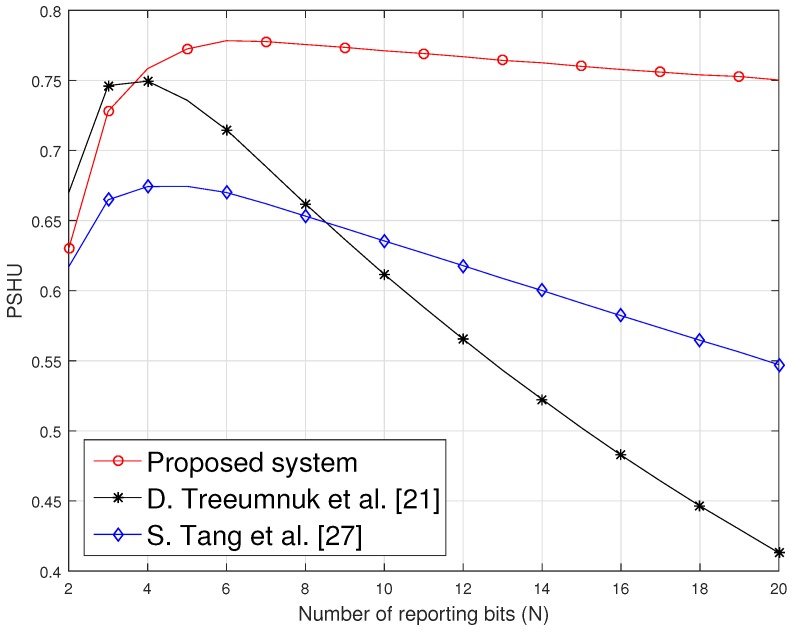
Probability of spectrum hole utilization (PSHU) vs. number of reporting bits *(N)*.

Figure 4 shows PSHU as a function of the numbers of reporting bits *N* for soft cooperative spectrum sensing scheme, where PSHU increases until *N* = 7, after that it starts decreasing because, when *N* increases, $T_{tr}$ of SUs goes down, which results in low PSHU. According to Equation (13), $P_{f}$ decreases when *N* increases, but, on the other hand, increasing *N* results in more reporting time for SUs to report their information to FC. So again, transmission time of SUs reduces due to increasing sensing and reporting time. Thus, there is a trade-off between PSHU and *N*, which gives maximum PSHU at the optimal value of *N* as shown in Figure 4.

The performance of the proposed method is influenced by $T_{I}$ in the form of probability of no interference ($P_{NI}$). As $P_{NI}$ is dependent on $T_{I}$ and $T_{tr}$, so $P_{NI}$ increases exponentially when the idle time of PU is greater than transmission time of SUs, but, on the other hand, probability of transmission time (ratio of transmission to whole time frame) decreases, which results in low PSHU ultimately. Similarly, $P_{NI}$ decreases exponentially until $T_{tr}$ becomes equal to PU’s idle time but again this results in low PSHU, although SUs have high transmission time probability at this point. Thus, our proposed method finds out the maximum value of PSHU in between these two minima of PSHU by optimizing the number of SUs and reporting bits. On the other hand, $P_{NI}$ in conventional methods discussed in [21,27] are not influenced by $T_{I}$ and $T_{tr}$, as in the proposed method. colorredThus, our proposed method outperforms the conventional methods by achieving the higher PSHU due to the exponential trend of $P_{NI}$.

**Figure 5:**
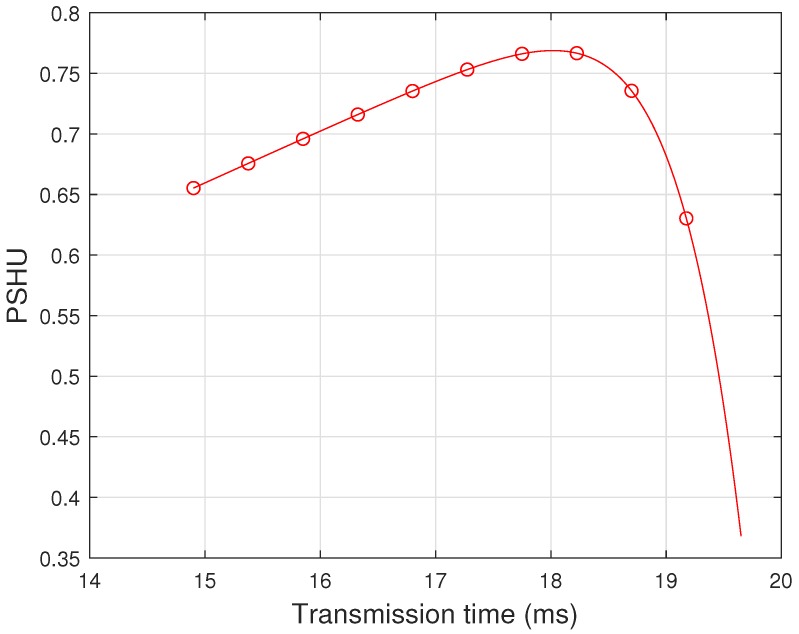
Probability of spectrum hole utilization (PSHU) vs. tranmission time ($T_{tr}$) of SUs.

A trade-off between PSHU and transmission time is shown in Figure 5, which shows that PSHU increases with $T_{tr}$ up to some specific value of $T_{tr}$ and beyond that it starts decreasing. This is because, when transmission time increases, it comes out with small time slots for sensing and reporting, so ultimately both probability of correct detection ($P_{CD}$) and probability of no interference ($P_{NI}$) start decreasing. Thus, there is a trade-off between PSHU and transmission time, which gives an optimal value of transmission time to achieve the maximum PSHU.

**Figure 6:**
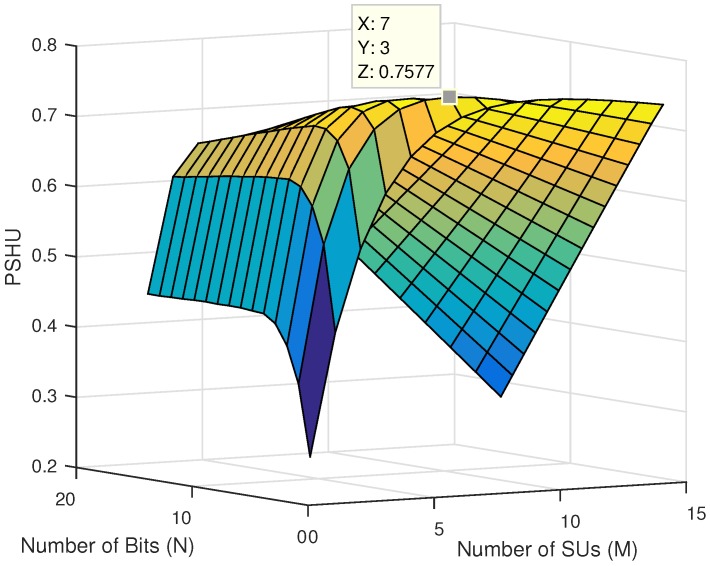
Joint optimization of the number of SUs and the number of bits to get the maximum PSHU.

Furthermore, the joint optimization of PSHU of the proposed system with respect to the number of SUs and the number of reporting bits is performed by simulations. Figure 6 shows that the maximum PSHU can be achieved by the optimal operating point that is obtained by the joint optimization of the number of SUs and the number of reporting bits.

## 7. Conclusions

This paper proposes a practical CR system and analyzes the PSHU under a soft cooperative spectrum sensing (CSS) scenario. In practical systems, the frame structure needs to be standardized and fixed and so does the length of the time-slot. High detection probability of primary users’ signal and high spectrum utilization by SUs can be achieved by adjusting the length of time slots of SUs inside the time frame. However, these achievements are not proportional to each other because an increase in sensing time for better detection of primary users’ signal reduces the transmission time, which causes a reduction of spectrum utilization. Similarly, SUs reporting in multiple bits form in cooperative spectrum sensing need longer reporting time, which also reduces the transmission time. Thus, a trade-off between PSHU and the number of SUs and the reporting bits is analysed in this paper, which shows the optimal number of SUs and the optimal number of reporting bits to achieve the maximum PSHU.
